# Long COVID Is Associated with Excess Direct Healthcare Expenditures Among Adults in the United States

**DOI:** 10.3390/healthcare13212704

**Published:** 2025-10-27

**Authors:** Rolake Neba, Lakshmi Sraddha Pedaprolu, Bryan Neba, Usha Sambamoorthi

**Affiliations:** 1 Health College of Pharmacy, University of North Texas, Fort Worth, TX 76107, USA; sraddha.pedaprolu@gmail.com (L.S.P.); usha.sambamoorthi@unthsc.edu (U.S.); 2 Independent Researcher, 4130 North Collins St., Arlington, TX 76005, USA; bryan.neba@lthcsolutions.com

**Keywords:** long COVID, healthcare expenditures, COVID

## Abstract

**Background:** Long COVID can lead to a considerable economic burden because of ongoing care for persistent symptoms such as fatigue, dyspnea, or cognitive dysfunction. However, systematic research quantifying healthcare expenditures associated with long COVID remains limited. **Objective:** This study estimated the excess total, payer, and out-of-pocket healthcare expenditures associated with long COVID among adults in the United States (US). **Methods:** This was a cross-sectional analysis on adults ≥18 years using 2022 Medical Expenditure Panel Survey (MEPS) data (N = 17,119; representing approximately 254 million adults). Economic burden was measured with (1) total, (2) payer, and (3) out-of-pocket expenditures by individuals and their families. Generalized linear models (GLMs) with gamma distribution and log link were utilized to estimate excess expenditures associated with long COVID after adjusting for age, sex, race and ethnicity, social determinants of health, health status, and lifestyle factors. **Results:** Overall, 7.0% of the population reported long COVID. Adults with long COVID exhibited higher total (USD 11,305 vs. USD 7162) and payer (USD 9983 vs. USD 6097) expenditures compared to those with no COVID. In a fully adjusted analysis, long COVID was associated with an excess of USD 4098 in total healthcare expenditures and USD 3705 in payer expenditures. We did not observe significant differences in out-of-pocket expenditures between those with long COVID and no COVID. **Conclusions:** Adults with long COVID had 1.5 times higher total healthcare costs compared to those without COVID. This study highlights the need for comprehensive strategies and policies to reduce the economic burden associated with long COVID.

## 1. Introduction

The World Health Organization defines long COVID as the continuation or development of new symptoms at least three months after an initial SARS-CoV-2 infection, lasting for at least two months with no alternative explanation [1]. Long COVID has become an emerging issue with substantial clinical and humanistic burden because of wide-ranging symptoms such as fatigue, dyspnea, cough, headache, chest and joint pains, smell and taste disturbances, cognitive and mental impairments, and gastrointestinal and cardiac issues [2]. Additionally, long COVID has been linked to the onset or exacerbation of other chronic conditions such as migraines, lung disease, autoimmune disease, and chronic kidney disease [2].

Globally, an estimated 65 million individuals are affected by long COVID [3]. In the United States (US), prevalence estimates range from as low as 1.3% in children [4] to as high as 32.2% among adults, ref. [5] depending on population and symptom definitions. Nationwide, among non-institutionalized civilian adults (i.e., individuals living in households and community settings rather than in institutions such as nursing homes, prisons, or long-term care facilities), the prevalence of long COVID has been estimated at 7% [6].

Beyond the high prevalence and clinical burden, long COVID can lead to the financial hardship of individuals and society through higher direct and indirect medical expenditures and diminished productivity. Several studies report significant economic impacts within the United States and internationally. A computational simulation model study revealed that the direct medical expenditures due to COVID may vary by age, sex, and long COVID duration, with an average total expenditure for an adult male being USD 9906 with an uncertainty range of USD 2637–USD 27,386 [7]. Furthermore, the same study reported that 95% (USD 9432) of these costs were due to productivity losses. David Cutler estimated the economic costs of long COVID in the United States (US) at USD 3.7 trillion, comprising lost quality of life (~USD 2.2 trillion), cost of lost earning (USD 997 billion), and spending on healthcare (USD 528 billion with USD 11,189 per capita costs (~USD 1566 direct healthcare expenditures) [8]. An analysis of the Census Household Pulse Survey shows that U.S. working-age adults lost an estimated USD 211 billion in earnings to long COVID in 2022, rising to USD 218 billion in 2023 [9]. In terms of direct healthcare expenditures, a retrospective study of insurance claims data found that those with long COVID had two times higher 6-month expenditures (USD 1182.8 vs. USD 2473.6) compared to the pre-diagnosis period [10]. Using Colorado state’s All-Payer Claims Database, researchers observed that adults with long COVID visited outpatient clinics and specialists more often in the year after diagnosis [11]. This uptick in service use implies considerably higher direct healthcare expenditures.

A population-based longitudinal Electronic Health Records study from the United Kingdom reported persistent higher expenditures even two years after long COVID diagnosis [12]. In this study, those with long COVID had nearly 1.5–2.0 times as much as expenditures as in age- and comorbidity-matched individuals during the pandemic [12]. A study from Israel reported that among individuals without long COVID, healthcare expenditures decreased from New Israeli Shekel-(NIS) 1400 to NIS 1021 four months post-infection. In contrast, expenditures among those with long COVID increased from NIS 2435 to NIS 2810 [13].

Although existing research from the US and elsewhere has highlighted the economic burden of long COVID, nation-wide representative studies that have examined the association of long COVID with healthcare expenditures at the individual level in the US is sparse. Such studies are critical to gain a comprehensive understanding of the economic burden of long COVID. Therefore, the objective of this study was to estimate the excess total, payer, and out-of-pocket direct healthcare expenditures associated with long COVID among adults in the U.S. The individual-level expenditure estimations are essential for guiding policymakers, payers, and employers in developing targeted strategies to mitigate the financial impact of long COVID on patients and the healthcare system.

## 2. Materials and Methods

### 2.1. Study Design and Data Source

This study utilized a cross-sectional design, obtaining data from the 2022 Medical Expenditure Panel Survey (MEPS), a nationally representative sample of civilian households in the U.S. The MEPS is designed to examine healthcare resource use and expenditures. The MEPS gathers information on which health services Americans use and how often, as well as the expenses associated with those services. Thus, healthcare expenditures are defined as payments for healthcare services received by individuals [14]. Most healthcare expenditures reported by MEPS respondents are associated with individual healthcare events. The MEPS is also a comprehensive source of information on access to care, preventive health, employment, prescribed medications, provider characteristics, social and health experiences, health-related quality of life, and more [15].

### 2.2. Analytical Sample

We included all adult participants aged 18 years and older who had complete data on COVID status, healthcare expenditures, and key covariates. Individuals were excluded if they had missing information on any of the following: age, sex, race/ethnicity, education, insurance coverage, income, or health status. Only respondents present throughout all rounds of the 2022 survey year were included to ensure complete expenditure data.

### 2.3. Measures

#### 2.3.1. Dependent Variables: Annual Total, Payer, and Out-of-Pocket Healthcare Expenditures

Total healthcare expenditures represent payments made for healthcare services across sites (hospital, outpatient, emergency room, and long-term care facilities), type (home health, dental, vision, prescription, and others), and payers (self/family Medicaid, Medicare, private insurance, Veterans Administration/CHAMPVA, TRICARE, other federal, state, and local non-governmental agencies, Workers’ compensation, and others). Payer expenditures were computed by subtracting out-of-pocket expenditures from total healthcare expenditures. Out-of-pocket expenditures included “any deductible, coinsurance, and copayment amount not covered by other sources, as well as payments for services and providers not covered by the person’s insurance or other sources” paid by patients or their families [16]. MEPS investigators collect information from the participants every round (lasting approximately 180 days) to reduce recall bias [16]. All expenditure variables represent total payments for healthcare services incurred during the calendar year 2022, regardless of the date of COVID-19 infection or symptom onset.

#### 2.3.2. Key Independent Variable: Long COVID vs. Acute COVID and No COVID

In the MEPS, household respondents were first asked if each household member had ever had COVID-19. Among those who answered “yes”, a follow-up question asked whether the members had symptoms lasting 3 months or longer that they did not have prior to having COVID-19. Long COVID was defined as a “yes” response to both questions, reflecting symptoms for ≥3 months post-COVID. Respondents who reported having COVID but without long-lasting symptoms were categorized as having “acute COVID,” and those who never had COVID were categorized as “no COVID.”

#### 2.3.3. Other Independent Variables

Additional explanatory variables included demographic characteristics such as age (18–44,45–54, 55–64, 65+), sex (male, female), race and ethnicity (non-Hispanic white (NHW), non-Hispanic Black (NHB), Hispanic and other race), social determinants of health—SDOH (education, poverty status in relation to federal poverty line (FPL), employment, health insurance coverage, marital status, and region), health status (number of chronic physical conditions, depression, anxiety), and lifestyle behaviors (physical activity, cigarette smoking frequency, and COVID vaccination status). Please see Table 1 for the categories in each of the characteristics.

#### 2.3.4. Statistical Methods

As the MEPS uses a complex survey design, all our analyses accounted for the clustering, stratification, and person weights. Statistically significant group differences by COVID-status categories were tested using Rao–Scott chi-squared tests. Unadjusted group differences in total, payer, and out-of-pocket expenditures were evaluated using t-statistics. Due to the unique characteristics of healthcare expenditures (non-negative, rightly skewed), we opted for a generalized linear model (GLM) with appropriate function and log link. As the ordinary least squares regression assumes normally distributed, constant-variance errors, a log transformation to expenditures is required for analytical robustness. However, log-transformed expenditures cannot be easily transformed to the original dollar metric. Therefore, we used a GLM with gamma distribution and log link [17]. Under this approach, direct re-transformation on the dollar scale is feasible and exponentiated regression coefficients can be interpreted as percent change [18]. In these models, we adjusted for age, sex, race and ethnicity, SDOH, health status, COVID vaccination status, and lifestyle behaviors. We report adjusted means, excess expenditures, and cost ratios by COVID status. Adjusted mean expenditures are model-based averages of a typical U.S. adult (as represented in the MEPS) in each COVID group after adjusting for age, sex, race/ethnicity, SDOH, number of chronic conditions, depression, anxiety, physical activity, smoking, and COVID-19 vaccination. Excess cost is the difference between each group’s adjusted mean and the no-COVID mean. Cost ratios (relative changes) were obtained by exponentiating the log link model coefficients (i.e., exp [β]). All estimates include 95% confidence intervals derived using the delta method and account for the MEPS design. Descriptive analyses were conducted using SAS 9.4 (SAS Institute Inc., Cary, NC, USA), and generalized linear models were created using Stata 17.0, MP, Parallel Edition (StataCorp LLC, College Station, TX, USA).

## 3. Results

Among US adults aged 18 or older (Table 1), 48.7% were females. NHW comprised the largest group (60.8%), while Hispanic and non-Hispanic Black groups represented 17.6% and 12.1%, respectively. Nearly one-third (35.8%) had a college degree, with 45.2% reporting were high income (≥400% FPL). Over one-third (36.6%) of participants reported multimorbidity (two or more chronic conditions). An overwhelming majority of adults (83.5%) received the primary series COVID vaccination.

Overall, 7.0% of US adults experienced long COVID, 43.7% had acute COVID, and 49.3% did not report any COVID (Table 2). A higher percentage of females (8.7% vs. 5.2%) than males reported long COVID. Long COVID prevalence was highest among those aged 45–54 and 55–64 (9.2% each) compared to 6.1% in the 18–44 group and 5.8% in those aged >65 years. Racial and ethnic differences were evident: non-Hispanic White individuals had the highest prevalence of long COVID (7.9%), followed by Hispanic (6.7%) and non-Hispanic Black individuals (4.5%).

Table 2 also displays the average total, payer, and out-of-pocket healthcare expenditures by COVID categories. Overall, the average total expenditures were USD 7673 (standard error (SE) = 190.3), including payer expenditures (USD 6605, SE = 176.3) and out-of-pocket spending (USD 1068, SE = 41.3). Adults with long COVID had notably higher total (USD 11,305 vs. USD 7162), payer (USD 9983 vs. USD 6097), and out-of-pocket (USD 1322 vs. USD 1065) expenditures compared to those without COVID.

Table 3 presents the findings from the multivariable GLM with gamma distribution and log link on total, payer, and out-of-pocket expenditures. Adults with long COVID had higher total and payer expenditures compared to those without COVID. When translated into percentages by exponentiating the regression coefficients, we observed that adults with COVID had 1.54 and 1.58 times greater total and payer expenditures as those of adults without COVID. After adjustment, adults with long COVID had the highest total expenditures. The adjusted mean was USD 11,641 (95% CI USD 9279–USD 14,004) for long COVID, USD 8361 (7656–9066) for acute COVID, and USD 7543 (6984–8103) for no COVID. When compared to no COVID, the excess total expenditures were +USD 4098 (95% CI USD 1619–USD 6578) for long COVID and +USD 818 (−USD 93 to USD 1728) for acute COVID. Most of the long COVID excess was borne by third-party payers (+USD 3705, 1442–5968), while out-of-pocket differences were small and not statistically significant (+USD 236, −95 to 566).

However, we did not observe a statistically significant difference in out-of-pocket expenditures by COVID categories.

To evaluate whether results were driven by multimorbidity, we conducted analyses stratified by multimorbidity (≥2 vs. 0–1 chronic conditions). For each outcome (total, third-party, OOP), we fit survey-weighted two-part models (logit for any spending; gamma with log link for positive spending) with the same covariates as the primary analysis and reported average marginal effects (dy/dx) for long COVID and acute COVID versus no COVID (interpreted as excess USD ).

Our results indicated that compared to no COVID, excess total expenditures for long COVID were USD 3737 (SE USD 1234; *p* < 0.01) among those with multimorbidity and USD 2603 (SE USD 1112; *p* < 0.05) among those no multimorbidity. Long COVID excess third-party spending was USD 4192 (SE USD 1356; *p* < 0.001) for the multimorbidity group and USD 1216 (SE USD 989; *p* < 0.05) for the no multimorbidity group. We did not observe statistically significant differences in OOP expenditures. Thus, our main findings are not explained by comorbidity burden alone. Please note that because two-part models are nonlinear and were fit independently, the model-based contrasts (excess USD) are not algebraically constrained to satisfy third-party + OOP = total.

## 4. Discussion

Overall, about 1 in 14 U.S. adults reported long COVID, highlighting the substantial population affected by ongoing symptoms. The estimated long COVID prevalence of 7.0% among US adults found in our study closely aligns with recent national data on non-institutionalized civilians. For example, the Census Household Pulse Survey found that the prevalence of long COVID among non-institutionalized adults was 7.5% during 7–19 June 2023 [19]. Similarly, an analysis of 2022 National Health Interview Survey (NHIS) data estimated that 6.9% of adults had experienced long COVID [20]. This similarity may reflect that both the MEPS and national surveys referenced restricted analyses to non-institutionalized, civilian populations. As such, our estimate is consistent with others, capturing the same population scope, though prevalence in institutionalized settings remains less well-characterized. Because the MEPS relies on self-reported COVID history, the proportion classified as ‘no COVID’ may be overestimated. CDC seroprevalence data indicate that by July–September 2022, 70.3% of U.S. adults had evidence of prior infection (22.6% infection-induced only, 47.7% hybrid immunity), which suggests that underreporting is likely [21].

In our study, adults with long COVID had higher healthcare expenditures compared to those without COVID, consistent with other studies reported in the literature [22]. Expenditures among individuals with acute COVID generally fell between those with long COVID and those with no COVID. Given the broad and persistent symptomatology of long COVID, affected individuals utilize a wide range of healthcare services including general practitioner visits, outpatient services, and emergency department visits [11]. Although COVID-19 was initially recognized as a respiratory illness, SARS-CoV-2 is now understood to affect multiple organ systems. This multisystem involvement is thought to be primarily mediated by immune activation and persistent inflammation rather than direct viral invasion [3]. In particular, endothelial dysfunction has emerged as a hallmark of long COVID [3]. Additionally, alterations in the size and stiffness of blood cells have been identified in individuals with long COVID, potentially impairing oxygen delivery and perpetuating organ dysfunction [3]. These biological disruptions may help explain the progression of existing chronic illnesses and the emergence of new conditions, many of which are not fully captured in administrative data or clinical records. Furthermore, these findings underscore that persistent symptoms, the worsening of existing chronic conditions, and the new onset of chronic conditions due to long COVID require ongoing healthcare needs long after the acute infection is resolved.

Research has consistently shown that individuals who develop long COVID often have more underlying chronic conditions than those who do not. Our study supports these findings, as individuals with multiple chronic conditions were more likely to experience long COVID. For instance, research has reported that long COVID patients had a higher Charlson comorbidity index (1.2 vs. 0.9), indicating a higher baseline health burden [23]. Although multimorbidity was more prevalent among individuals with long COVID, our findings suggest that it does not fully explain the observed differences in healthcare expenditures across groups. Chronic conditions likely compound long COVID’s impact, as managing long COVID in addition to multiple chronic illnesses may lead to frequent appointments, medication adjustments, and potential complications. It can be challenging to disentangle long COVID-specific costs from overall healthcare costs in such cases; however, our study revealed that individuals with comorbidities had a higher percentage of total healthcare expenditures compared to those without comorbidities. Long COVID patients may need to consult multiple specialists due to various chronic conditions, which can lead to prescribing several medications, increasing the risk of fragmented care and polypharmacy [24]. Multidisciplinary clinics streamline care delivery and can mitigate the cost effects of long COVID. The Agency for Healthcare Research and Quality has awarded grants to support such clinics, aiming to provide comprehensive, coordinated, and person-centered care for people with long COVID, particularly in communities disproportionally affects by the impacts of long COVID [25].

In our analysis, we did not detect a statistically significant difference in annual out-of-pocket medical spending between individuals with long COVID and those without COVID. Despite significantly higher total and payer expenditures among adults with long COVID, this finding suggests that much of the financial burden is absorbed by third-party payers—including private and public insurers—rather than falling directly on patients. This apparent discrepancy arises because out-of-pocket expenditures represent a relatively small and highly variable component of total healthcare costs. In the U.S., most COVID-19-related expenditures are covered by insurance, which concentrates cost differences within payer spending rather than patient OOP contributions. These results align with the structure of U.S. health insurance systems, where deductibles and copayments typically represent only a fraction of total costs. However, out-of-pocket expenses represent just one facet of the overall financial burden. As highlighted in the introduction, indirect costs—such as lost productivity, reduced work capacity, and caregiving demands—are substantial [9]. To fully capture the economic impact of long COVID, future research should incorporate both direct and indirect cost measures and examine how evolving care pathways and treatment approaches influence long-term patient financial responsibilities.

Our study benefits from a large, nationally representative sample of U.S. adults and the detailed expenditure information provided by the MEPS. This allowed for a comprehensive understanding of the economic burden of long COVID on both individuals and third-party payers. Furthermore, the study adjusts for a wide range of factors that may influence healthcare expenditures, such as demographic factors, socioeconomic status, and lifestyle factors. However, several limitations should be considered. The MEPS collects self-reported data on health conditions, socioeconomic factors, and lifestyle behaviors, which may be subject to recall bias or reporting inaccuracies. As an observational study with a cross-sectional design, causality cannot be established. Because temporality between long COVID onset and healthcare utilization cannot be determined, higher expenditures may partly reflect pre-existing morbidity or healthcare needs unrelated to long COVID itself. The use of data from only one calendar year (2022) also limits the ability to assess changes in long COVID-related expenditures over time; future studies should incorporate multi-year analyses to evaluate trends. Because we used the annual consolidated file, we could not assess the within-year timing of COVID onset, symptoms, or expenditures captured at the quarterly level; COVID status capture may vary by quarter. This limits inferences on temporality and may introduce misclassification. Despite extensive covariate adjustment, unmeasured confounding remains possible, as variables such as COVID severity, pre-infection healthcare utilization, vaccination timing, and chronic disease severity and duration were unavailable. Additionally, the classification of long COVID relied on self-reporting, which may lead to misclassification; future research should validate diagnoses using clinical records or standardized symptom inventories. Furthermore, the MEPS does not collect information on specific symptom clusters (e.g., fatigue, cognitive dysfunction, respiratory issues due to COVID), which limits our ability to assess whether different long COVID phenotypes are associated with varying healthcare cost burdens. Because the MEPS includes only non-institutionalized survivors, we could not capture the highest-severity outcomes such as institutionalization or death, which may lead to underestimation of the true economic burden. Finally, our analysis was limited to direct healthcare expenditures and did not include indirect costs such as absenteeism, lost productivity, or societal costs. As such, our estimates reflect a healthcare system and payer perspective rather than the total economic burden of long COVID.

## 5. Conclusions

Adults with long COVID incurred 1.5 times higher healthcare expenditures compared to those without COVID, with most of the burden on insurers and payers. These findings underscore the substantial and persistent impact of long COVID on the U.S. healthcare system. Policymakers, payers, and healthcare providers should prepare for the long-term resource implications, particularly for populations with multimorbidity or other social vulnerabilities. Proactive investments in coordinated care models, such as multidisciplinary clinics and patient-centered medical homes, are essential to managing both the clinical complexity and economic costs associated with long COVID.

## Figures and Tables

**Table 1:** Characteristics of adults (age > 18 years). Medical Expenditure Panel Survey, 2022.

		N	Wt. N	Wt. %
ALL		17,119	253,953,449	100.0
**Sex**				
	Female	9273	130,322,477	51.3
	Male	7846	123,630,972	48.7
**Age in Years**				
	18–44 years	6391	116,360,187	45.8
	45–54 years	2588	39,497,330	15.6
	55–64 years	3058	40,906,863	16.1
	65+ years	5082	57,189,069	22.5
**Race and Ethnicity**				
	NHW	9802	154,393,273	60.8
	NHB	2459	30,764,187	12.1
	Hispanic	3405	44,705,698	17.6
	Other race	1453	24,090,291	9.5
**Education**				
	LT high school (HS)	2533	31,355,031	12.3
	HS	4883	70,934,403	27.9
	Some college	3655	58,957,176	23.2
	College	5914	90,793,816	35.8
**Employment**				
	Employed	10,300	170,622,088	67.2
	Not employed	6801	82,962,016	32.7
**Poverty Status**				
	Poor	2643	26,569,126	10.5
	Near poor	2982	37,645,513	14.8
	Middle income	4744	74,511,037	29.3
	High income	6750	115,227,772	45.4
**Health Insurance**				
	Private	10,299	170,378,707	67.1
	Public	5494	64,336,122	25.3
	None	1326	19,238,620	7.6
**Marital Status**				
	Married	8175	128,097,253	50.4
	Widowed	1497	16,387,902	6.5
	Div/separated	2800	32,651,435	12.9
	Never married	4646	76,804,207	30.2
**Region**				
	Northeast	2723	44,016,175	17.3
	Midwest	3436	51,764,599	20.4
	South	6594	98,500,842	38.8
	West	4366	59,671,832	23.5
**Chronic Conditions**				
	Multimorbidity	7493	92,907,115	36.6
	No Multimorbidity	9626	161,046,334	63.4
**Depression**				
	Depression	1639	22,679,990	8.9
	No depression	15,480	231,273,459	91.1
**Anxiety**				
	Anxiety	1865	26,163,391	10.3
	No anxiety	15,254	227,790,058	89.7
**Cigarette Use**				
	Every day	1356	18,696,503	7.4
	Some days	687	9,455,020	3.7
	Not at all	14,935	223,413,747	88.0
**Physical Activity**				
	GE 5 times/week	8541	131,009,373	51.6
	Other	8386	119,814,866	47.2
**COVID Vaccination**				
	COVID vaccine	14,323	210,726,944	83.5
	No COVID vaccine	2706	41,605,713	16.5

Note: Based on 17,119 adults aged 18 or older from the Medical Expenditure Panel Survey with no missing values for COVID status. Missing data in the variables employment, education, marital status, cigarette use, physical activity, and COVID vaccination are not presented in the table. Div: divorced; GE: greater than or equal to; NHW: non-Hispanic White; NHB: non-Hispanic Black.

**Table 2:** N and weighted row percentages of adults (age > 18 years) by COVID categories. Medical Expenditure Panel Survey, 2022.

		Long COVID		Acute COVID		No COVID			
		N	Wt. %	N	Wt. %	N	Wt. %	Chi-Sq	*p*-Value
ALL		1196	7.0	6984	43.7	8939	49.3		
**Sex**								64.1	<0.001
	Female	801	8.7	3743	43.7	4729	47.6		
	Male	395	5.2	3241	43.7	4210	51.1		
**Age in Years**								222.0	<0.001
	18–44 years	398	6.1	3071	48.6	2922	45.3		
	45–54 years	236	9.2	1143	47.3	1209	43.5		
	55–64 years	274	9.2	1172	41.1	1612	49.7		
	65+ years	288	5.8	1598	33.0	3196	61.2		
**Race and Ethnicity**								107.23	<0.001
	NHW	766	7.9	4236	46.0	4800	46.1		
	NHB	122	4.5	743	34.7	1594	60.8		
	Hispanic	243	6.7	1409	43.3	1753	50.0		
	Other race	65	4.6	596	41.1	792	54.3		
**Education**								167.9	<0.001
	LT high school (HS)	155	5.6	852	36.3	1526	58.1		
	HS	346	7.5	1767	39.0	2770	53.5		
	Some college	319	8.2	1432	42.1	1904	49.8		
	College	370	6.3	2896	51.3	2648	42.4		
**Employment**								307.3	<0.001
	Employed	756	7.1	4852	48.8	4692	44.0		
	Not employed	439	6.7	2126	33.2	4236	60.1		
**Poverty Status**								159.1	<0.001
	Poor	179	6.8	795	32.3	1669	61.0		
	Near poor	234	7.9	987	36.5	1761	55.6		
	Middle income	357	7.9	1918	42.3	2469	49.9		
	High income	426	6.2	3284	49.6	3040	44.2		
**Health Insurance**								231.9	<0.001
	Private	740	7.1	4757	48.6	4802	44.4		
	Public	379	6.9	1816	34.9	3299	58.2		
	None	77	6.5	411	30.0	838	63.5		
**Marital Status**								156.6	<0.001
	Married	604	7.6	3720	47.8	3851	44.6		
	Widowed	116	8.3	414	29.6	967	62.1		
	Div/separated	234	8.0	981	37.4	1585	54.5		
	Never married	242	5.3	1869	42.6	2535	52.2		
**Region**								23.3	<0.001
	Northeast	185	6.3	1179	46.4	1359	47.3		
	Mid-west	266	8.4	1489	45.0	1681	46.6		
	South	433	6.6	2483	41.4	3678	52.0		
	West	312	6.9	1833	44.4	2221	48.7		
**Chronic Conditions**								90.9	<0.001
	Multimorbidity	654	9.4	2683	38.9	4156	51.7		
	No multimorbidity	542	5.6	4301	46.5	4783	47.9		
**Depression**								33.6	<0.001
	Depression	186	11.5	666	44.5	787	44.0		
	No depression	1010	6.6	6318	43.6	8152	49.8		
**Anxiety**								71.5	<0.001
	Anxiety	222	12.1	828	48.0	815	39.9		
	No anxiety	974	6.4	6156	43.2	8124	50.4		
**Tobacco Use**								66.9	<0.001
	Everyday	103	7.9	378	31.2	875	60.8		
	Some days	46	6.7	231	37.2	410	56.1		
	Not at all	1038	6.9	6320	45.1	7577	47.9		
**Physical Activity**								6.1	0.193
	GE 5 times/week	565	6.8	3551	43.8	4425	49.4		
	Other	618	7.2	3362	43.8	4406	48.9		
**COVID Vaccination**								6.2	0.045
	COVID vaccine	956	6.7	5890	44.2	7477	49.2		
	No COVID vaccine	234	8.4	1073	42.1	1399	49.5		
**Expenditures**		**Mean USD**	**SE**	**Mean USD**	**SE**	**Mean USD**	**SE**		
	Total	11,305.3	871.9	7668.8	306.8	7161.9	226.0		
	Payer	9983.3	853.4	6638.7	295.0	6097.1	201.0		
	Out-of-pocket	1322.0	132.3	1030.1	35.8	1064.8	70.6		

Note: Based on 17,119 adults aged 18 or older from the Medical Expenditure Panel Survey with no missing values for COVID status. Missing data in the variables employment, education, marital status, tobacco use, physical activity, and COVID vaccination are not presented in the table. Significant group differences in COVID status categories were derived using Rao–Scott chi-squared tests. Div: divorced; GE: greater than or equal to; NHW: non-Hispanic White; NHB: non-Hispanic Black.

**Table 3:** Adjusted mean, excess expenditures, cost ratios, and 95% confidence intervals of COVID status from survey generalized linear models with gamma distribution and log link total, payer, and out-of-pocket expenditures among adults (age > 18 years). Medical Expenditure Panel Survey, 2022.

		Adjusted Means (95% CI)	*p*-Value	Excess USD (95% CI)	Cost Ratios (95% CI)
**Total Expenditures**					
**COVID Categories**					
	Long COVID	USD 11,641 (9279–14,004)	0.001	USD 4098 (1619–6578)	1.54 (1.24–1.93)
	Acute COVID	USD 8361 (7656–9066)	0.078	USD 818 (−93–1728)	1.11 (0.99–1.24)
	No COVID (Ref)	USD 7543 (6984–8103)		Ref	Ref
**Payer Expenditures**					
**COVID Categories**					
	Long COVID	USD 10,135 (7934–12,337)	0.001	USD 3705 (1442–5968)	1.58 (1.25–1.99)
	Acute COVID	USD 7289 (6607–7972)	0.050	USD 859 (1.37–1716)	1.13 (1.00–1.28)
	No COVID (Ref)	USD 6431 (5933–6928)		Ref	Ref
**Out-of-Pocket Expenditures**					
**COVID Categories**					
	Long COVID	USD 1348 (1049–1648)	0.162	USD 236 (−95–566)	1.21 (0.94–1.56)
	Acute COVID	USD 1140 (1054–1227)	0.716	USD 28 (−122–177)	1.02 (0.90–1.17)
	No COVID (Ref)	USD 1112 (973–1251)		Ref	Ref

Note: Based on 17,119 adults aged 18 or older from the Medical Expenditure Panel Survey with no missing values for COVID status. Missing data indicators for the variables employment, education, marital status, tobacco use, physical activity, and COVID vaccination were included in the regressions. Covariates: sex, age, race and ethnicity, education, employment, poverty status, insurance (medical and prescription), US region, number of chronic conditions, depression, anxiety, physical activity, cigarettes per day, and COVID-19 vaccination. 95% CIs were derived using the delta method. Ref: reference group.

## Data Availability

No restrictions apply. Data was obtained from the 2022 Medical Expenditure Panel Survey (MEPS): https://www.meps.ahrq.gov/data_stats/download_data/pufs/h243/h243doc.pdf (accessed on 27 June 2025).

## Abbreviations

The following abbreviations are used in this manuscript:

MEPS	Medical Expenditure Panel Survey
GLM	Generalized Linear Models
NHW	Non-Hispanic White
NHB	Non-Hispanic Black
SDOH	Social Determinants of Health
FPL	Federal Poverty Line
